# The Effect of Moral Congruence of Calls to Action and Salient Social Norms on Online Charitable Donations: A Protocol Study

**DOI:** 10.3389/fpsyg.2018.01913

**Published:** 2018-10-26

**Authors:** Nikola Erceg, Matthias Burghart, Alessia Cottone, Jessica Lorimer, Kiran Manku, Hannah Pütz, Denis Vlašiček, Manou Willems

**Affiliations:** ^1^Department of Psychology, Faculty of Humanities and Social Sciences, University of Zagreb, Zagreb, Croatia; ^2^Department of Clinical Psychological Science, Maastricht University, Maastricht, Netherlands; ^3^Department of Psychology, University of Leicester, Leicester, United Kingdom; ^4^Department of Psychiatry, University of Oxford, Oxford, United Kingdom; ^5^Department of Social Policy and Intervention, University of Oxford, Oxford, United Kingdom

**Keywords:** charitable behavior, moral foundations, moral identity internalization, social norms, Facebook data

## Abstract

Online advertising is an important tool that can be utilized by charities to elicit attention and funding. A critical examination of advertisement strategies is thus necessary to increase the efficacy of fundraising efforts. Previous studies have shown that individuals’ moral views and perceptions of social norms can play important roles in charitable behavior. Thus, the current protocol describes a study to examine whether framing charitable advertisements in line with participants’ morality and increasing the salience of descriptive social norms increases subsequent charitable behavior. We describe experimental, online methods, whereby participants are provided with a framed call-to-action and normative information within a custom-developed application or existing survey platform. Furthermore, in the exploratory fashion, we discuss the possibility of collecting participants’ Facebook data and predicting moral profiles from this data. If there is an increased rate of donations as a result of moral compatibility and/or increased norm salience, charities can leverage this knowledge to increase the donations by tailoring their campaigns in a more appealing way for their prospective donors. Moreover, if it turns out to be possible to predict one’s moral profile from Facebook footprints, charities can use this knowledge to find and target people that are more likely to support their cause. However, this introduces important ethical questions that are discussed within this protocol.

## Introduction

Charities often provide a vital service for marginalized and vulnerable people in society. Given that individual charitable giving now contributes the largest proportion of income for all registered charities in the United Kingdom (The National Council for Voluntary Organisations [NCVO], 2017), it is vital that charities maximize the effectiveness of their campaigns. A promising avenue for increasing the efficiency of charitable fundraising with little or no additional cost are campaigns in the digital sphere, a rapidly growing platform for philanthropy. In various reports, MacLaughlin (2012, 2014, 2017) has shown that online giving has been steadily growing in past years. For example, between 2013 and 2014, it increased by 8.9% (MacLaughlin, 2014). Furthermore, from 2015 to 2016, online giving increased by 2.8% in the United Kingdom and 7.9% in the United States (MacLaughlin, 2017). The following protocol suggests a method to study whether tailored appeals and salient social norms can be utilized to increase individual charitable behavior. The findings based on this protocol could significantly aid the online marketing strategies of charitable organizations.

### The Determinants of Charitable Behavior

In this protocol, charitable behavior (CB) refers to the measurable actions of supporting a charity through donating money or time. To construct an intervention that influences CB, it is first necessary to identify its potential determinants. CB has often been examined in the context of the Theory of Planned Behavior (TPB; Ajzen, 1985, 1991). In the TPB, the immediate causes of any behavior are (1) intentions to perform that behavior and (2) the actual control one has over performing it. In turn, behavioral intentions result from individuals’ attitudes, social norms and perceived behavioral control. Indeed, some studies found that these TPB variables are good predictors of CB. For example, van der Linden (2011) found that attitudes, perceived behavioral control, past behavior and moral norms significantly predicted charitable giving intentions. Smith and McSweeney (2007) reported similar results, but identified injunctive norms as an additional predictor of charitable giving intentions. Thus, one way to increase CB is to influence one or more of its immediate causes, such as changing attitudes and referencing a social norm.

#### Changing Behavior by Changing Attitudes

An intervention aimed at increasing CB may focus on changing people’s attitudes toward CB. One promising way of doing this is by using the assumptions of Regulatory Fit Theory (Higgins, 2005). According to Regulatory Fit Theory (RFT), it is possible to increase the effectiveness of a persuasive appeal by framing the arguments of a persuasive message in a way that fits one’s psychological characteristics. This could include motivational orientation (Updegraff et al., 2007), personality (Hirsh et al., 2012) or moral characteristics (Feinberg and Willer, 2013). Regulatory fit is hypothesized to shift attitudes through three intertwined mechanisms: by making the recipient ‘feel right’ during the message reception, by increasing the recipient’s strength of engagement with the message, which contributes to processing fluency, and by influencing elaboration likelihood (Cesario et al., 2008).

What psychological characteristics are most relevant in the context of CB? It seems that an individual’s morals play a significant role. For example, Smith and McSweeney (2007) and van der Linden (2011) showed that personal moral norms are one of the strongest predictors of CB. Furthermore, Aquino and Reed (2002) moral identity internalization, or degree to which moral traits are central to one’s self-concept, has been shown to influence: (a) type of charitable donations (time versus money), (b) donation intentions, (c) actual donations, and (d) emotions experienced during donations. Those who feel that morality is central to their self-concept: (a) prefer donating time instead of money, (b) show greater intentions to donate money, (c) are willing to actually donate more money, and (d) experience more positive donation related emotions than those who are lower on moral identity (Reed et al., 2007; Winterich et al., 2012). This evidence makes a strong case for the importance of individual morals in predicting CB.

Therefore, following from the RFT, it can be hypothesized that a person’s attitudes would change if a persuasive message was congruent with his/her moral views. One of the most influential and extensive theories that describe individuals’ moral systems is the Moral Foundations Theory (MFT; Haidt and Joseph, 2004; Haidt and Graham, 2007; Graham et al., 2011, 2012). MFT postulates that five different, innate, moral foundations provide a “first draft” of moral intuitions; but these intuitions can also be revised through exposure to social context and culture (for a detailed review, see Graham et al., 2012). The five moral foundations proposed by MFT are Care/Harm, Fairness/Cheating, Loyalty/Betrayal, Authority/Subversion, and Sanctity/Degradation (Graham et al., 2012). According to the theory, differences in these five foundations are responsible for differences in morality across individuals and cultures.

The first two foundations are called the *individualizing* *foundations*, meaning that they emphasize inter-individual relations. Individuals scoring highly on these foundations are primarily sensitive to possible cruelty, unfairness, and inequality when making moral judgments (Graham et al., 2012). In contrast, the last three foundations are called the *binding foundations*, which bind individuals into communities. According to the MFT, those scoring highly on these foundations are primarily sensitive to social community, hierarchical relations, and physical and spiritual purity when making moral judgments. Graham et al. (2009) found that politically liberal individuals primarily endorsed and used individualizing moral foundations when making judgments (i.e., Care/Harm and Fairness/Cheating), whereas conservatives endorsed and used all five foundations more equally. These correlational patterns between morality and political ideology have been shown to be stable across cultures (e.g., Bobbio et al., 2011; Graham et al., 2011; Kosugi et al., 2014). This suggests at least two distinct, universal moral foundations profiles: a liberal and a conservative one.

Recent studies have examined the influence of (in)congruence of messages and individuals’ moral foundations on attitudes toward CB, charitable intentions and actual CB. For example, Feinberg and Willer (2013) showed that framing messages about the environment in terms of sanctity, rather than only care, shifted conservatives’ attitudes in a pro-environmental direction. Building on this, Wolsko et al. (2016) showed that the attitude change was also accompanied by increased donations to pro-environmental causes. Additionally, the congruence between individuals’ moral foundations and both the charity cause and persuasive calls-to-action has been shown to increase donation intentions and donations, but only for those individuals high on moral identity internalization (Winterich et al., 2012; Nilsson et al., 2016).

Despite this, previous studies have not explicitly contrasted charitable causes and calls-to-action in order to see, for example, whether congruent calls-to-action can have a positive impact on CB even if the charity cause is not in line with one’s moral foundations (e.g., conservatives donating to a charity supporting immigration). Therefore, studying attitudes and subsequent behavior change in response to the (in)congruence between persuasive appeals of differing charity causes and individuals’ moral foundations is a promising research avenue.

#### Changing Behavior by Changing Social Norms

In addition to changing attitudes toward CB, a fruitful approach may be to influence perceptions of social norms about CB. Social norms are the perceived rules of a community or group that dictate desired behavior (Kandori, 1992). Although some studies show that norms are the weakest of the TPB predictors (see Armitage and Conner, 2001), others have pointed out the need to distinguish between several types of norms before assessing their contribution to behaviors. Specifically, three distinct types of normative influences have been identified. First, norms can be *injunctive*, representing the information about what most others approve or disapprove. Second, norms can be *descriptive*, conveying information about what most others actually do (Cialdini et al., 1990). Finally, personal injunctive norms, or moral norms can be defined as *individual internalized moral rules* (Smith and McSweeney, 2007; van der Linden, 2011). Thus far, several studies have investigated the role of different types of norms on prosocial behavior, showing mixed results (e.g., Shang and Croson, 2009; van der Linden, 2011).

Several studies showed that moral and injunctive norms significantly predicted charitable intentions, after controlling for attitudes, perceived behavioral control and past behavior (Smith and McSweeney, 2007; van der Linden, 2011). However, in these studies, descriptive norms did not have a significant influence on charitable giving. In contrast, other studies have shown that descriptive norms can be significant determinants of CB and prosocial behavior in general. For example, Shang and Croson (2009) demonstrated that providing individuals with information about the amount of money that others donated, influenced the amounts donated in public radio fundraising, both immediately and in renewals the year after. Moreover, Croson et al. (2009) showed that the effect of providing social information in such a way on donations to public radio is fully mediated by changes in the perception of descriptive social norms. It is possible that these mixed findings are the result of differences in the saliency of norms that were used in those studies.

A norm must be salient to be efficient in changing behavior (Cialdini et al., 1990). This may explain why descriptive norms did not influence CB in some of the previous studies: CB is often performed privately (Smith and McSweeney, 2007). Because CB is often performed privately, individuals may not have an accurate sense of the extent to which other people engage in charitable action. In other words, descriptive norms may be ineffective in the context of individual CB because they are not salient enough. Therefore, providing explicit information about the behavior of others, and thus making descriptive norms about CB salient, is hypothesized to be a useful approach to changing perceptions about descriptive social norms, and consequently CB itself.

### Leveraging Social Networks to Foster Charitable Behavior

The percentage of CB conducted online grows every year (e.g., MacLaughlin, 2017). As such, it would be beneficial for charities to take advantage of this and make their online fundraising campaigns more efficient. For example, if charities could target interested potential donors more precisely and approach them with tailored, congruent calls-to-action, this could significantly improve their fundraising outcomes.

One way to target potential donors more precisely is by using big data produced by social networks. When browsing social networks and engaging in behaviors on those networks, people leave digital footprints. Previous research has shown that these footprints can be predictive of different psychological characteristics. Kosinski and his colleagues have conducted multiple studies exploring the potential uses of digital footprints created on social media sites to identify users’ psychological characteristics (e.g., Quercia et al., 2011; Kosinski et al., 2013, 2014; Youyou et al., 2015).^1^

Their research shows that it is possible to predict people’s personality trait scores (e.g., openness and extraversion) based on their digital footprints – specifically, Facebook *likes*. In fact, these predictions can be as accurate as those made by human judges, such as colleagues, friends or spouses (Youyou et al., 2015). Other researchers have demonstrated that it is possible to use social media data to predict various other characteristics. For example, Conover et al. (2011) demonstrated that people’s political alignment can be predicted from their Twitter data with 90.8% accuracy. Furthermore, since an individual’s different psychological properties are reflected in his/her digital footprints, it is possible that these footprints could be used to predict the individual’s moral foundations. If so, it would be possible for charities to directly target people who are more likely to support their cause and become donors, while avoiding those who are less likely to support that specific cause.

However, it has to be noted that the future of data collection and advertising on Facebook is questionable, both pragmatically and from an ethical standpoint. From a practical point of view, there are two limitations: declining use and more stringent data sharing policies. For example, a Pew Research Center^2^ study completed in 2018 found that Facebook is no longer the most popular online platform among teens, with only half of teens reporting using it. In addition, mostly due to the Cambridge Analytica scandal ^3^, Facebook has made it’s data sharing policies much more stringent. In practice, they currently do not allow new applications to collect users’ data for research purposes. Moreover, the scandal echoed extremely negatively among its users, decreasing users’ trust toward the company. For example, a survey from the Ponemon Institute (2018) found that between 2017 and 2018, the percentage of people who believed that Facebook was committed to privacy dropped by 52 percentage points. Therefore, users may be reluctant to give away their information, even for scientific purposes.

However, as will be described in the protocol, the collection of Facebook data and prediction of moral views from it is completely optional and constitutes the exploratory part of this protocol. It is perfectly possible to skip this part and follow only the confirmatory part of the protocol. This would significantly simplify the procedure, while still potentially providing theoretically and practically meaningful findings.

## Aims and Hypotheses

The project has five aims:

(i) to investigate the effect of morally (in)congruent calls-to-action and salient descriptive social norms on CB;
(ii) to explore whether these effects occur when the charity’s cause is not aligned with a person’s moral profile;
(iii) to examine whether the effects of (in)congruent calls-to-action are moderated by participants’ moral internalization scores;
(iv) to investigate whether attitudes toward CB mediate the effect of (in)congruent calls-to-action on CB and whether descriptive social norms mediate the effect of normative information on CB;
(v) to investigate whether individuals’ moral foundations can be predicted based on their Facebook behavior.

**FIGURE 1:**
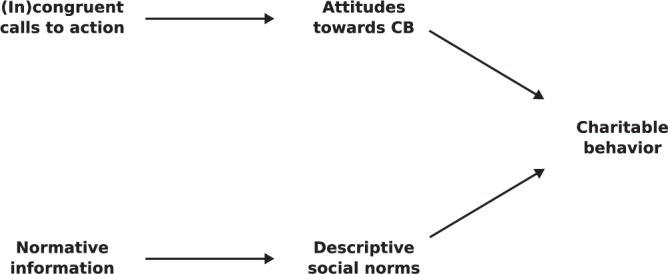
Schematic representation of the proposed effects between (in)congruent calls-to-action, normative information, attitudes toward CB, descriptive social norms and CB.

In previous studies, calls-to-action congruent with one’s moral foundations increased CB (Winterich et al., 2012). Therefore, we hypothesize that morally congruent calls-to-action will have a greater positive impact on CB compared with morally incongruent and neutral calls-to-action, regardless of whether the charity cause itself is in line with one’s moral foundations. Furthermore, we hypothesize that providing participants with normative information, thus making the descriptive norm salient, will significantly affect CB. Additionally, we expect that the impact of morally congruent calls-to-action will be greater in individuals with high moral internalization compared to individuals with low moral internalization. Also, in line with the assumptions of TPB (Ajzen, 1985, 1991), we expect that the effects of calls-to-action congruence on CB will be mediated by attitudes toward CB, and that the effect of normative information on CB will be mediated by the perception of descriptive social norms. Finally, although this part of the research would be purely exploratory, we expect to be able to predict some of the participants’ moral foundations by his/her Facebook behavior. The proposed effects are shown in the Figure 1.

## Materials and Equipment

### Participants

The number of participants needed when conducting the study according to this protocol mostly depends on the desired effect size we would like to be able to detect and the statistical power we would like to achieve. For example, using the multivariate analysis of variance, if we would like to achieve a power of at least 1−β = 0.90 to detect small effects (*f*^2^ = 0.02) with the probability of *Type I* error set at α = 0.05, we would need a sample size of *N* = 439 as calculated using *G^∗^Power 3* (Faul et al., 2007). However, given that it is expected that researchers will have to omit some participants whose scores do not meet the criteria for individualizing and binding groupings which will be determined from the data [due to calls-to-action not being (in)congruent enough with their moral views], it would be advisable to further increase the number of participants by 20%. Therefore, the ideal final sample would be around *N* = 527. Of course, if one would, for example, like to study the effects of (in)congruent calls-to-action and norms on donations to two different charity causes separately (e.g., those hypothesized to be more in line with liberals’ and conservatives’ values), one would need to double this number. On the other hand, if one is willing to the accept somewhat lower power of 1−β = 0.80, that is usually accepted in psychological research, one would need the sample size of *N* = 344, or around *N* = 412 if one decides to increase it by 20%.

The recruitment of participants can take place online. For example, in order to obtain a mixture of conservative and liberal participants, the link to the survey can be disseminated through different conservatively and liberally oriented Facebook groups (see Table A1 in Appendix A). Other online platforms where it is relatively easy to approach the users of different ideological positions can be also utilized, such as Reddit, Twitter etc.

### Facebook App

In this part we will describe the implementation of the survey and data collection using the custom made Facebook app. The Facebook app has several advantages over more traditional online surveys. First, it offers the possibility to customize the procedure and randomize some of its steps, which will be a useful feature for this study. Second, it allows automatic calculation of participants’ scores and instant customized feedback for each participant, which acts as a motivator for participation. Third, it allows us to collect some of the Facebook data that will be crucial for our exploratory part of the study (i.e., predicting the moral foundations). Finally, it facilitates the dissemination of the study by allowing the participants to share their scores and invite others to participate. A clickable URL will take participants to a custom-made Facebook app that will host the survey. The survey will include measurements of moral foundations, moral identity internalization, attitudes toward CB, descriptive social norms and CB in addition to relevant (in)congruent calls-to-action for the charities. The charities will be used based on the pre-study results. The wireframe of an app is presented in Figure A1 in Appendix A.

However, as noted in the introduction, it is perfectly possible to conduct the study based on this research protocol without collecting Facebook data or developing the custom Facebook app. In this case one could simply use one of the many existing research platforms to create the survey and disseminate it over different online platforms.

### Pre-study

For the pre-study purposes, all the measures except the MFQ were translated into German, Dutch, and Italian (i.e., the calls-to-action, Moral Identity Internalization Scale and the measures of attitudes and social norms) The translated copies of the MFQ were already publicly available on the original authors’ website^4^. The translation of the other scales was done using a forward and back translation method, in which a native speaker first translated the original version of the measure from English to the target language (German/Dutch/Italian), trying to keep conceptual rather than literal meaning. Next, a separate bilingual speaker translated the translation back to English. Finally, the group of authors reviewed and compared the translations. Consensus among the authors was reached before continuing with pilot testing.

After translation of the materials, we conducted the pre-study (*N* = 50). There were several goals of this pre-study. First, we confirmed that there were no ambiguities and confusions regarding the translations and instructions. Second, the pilot study was used to check the appropriateness of the calls-to-action which are the main independent variable. Specifically, we wanted to receive feedback on whether our calls-to-action were perceived as being based on the individualizing and binding moral foundations. To do this, we first briefly familiarized our participants with the MFT and each of the moral foundations. Thereafter, we asked them to estimate on a seven-point scale the degree to which each of the calls-to-action rely on each of the five foundations. As we specifically created our calls-to-action using words from the *Moral Foundations Dictionary* (it can be found online: http://moralfoundations.org/othermaterials), we expected that our individualizing calls will be estimated to rely mostly on the Care/Harm and Fairness/Cheating foundations. Accordingly, we expected binding calls to be estimated to rely more heavily on the Loyalty/Betrayal, Authority/Subversion, and Sanctity/Degradation foundations.

Third, we wanted to examine whether different charities were perceived as being more acceptable for liberals, more acceptable for conservatives or universally acceptable. Specifically, we listed brief descriptions of 12 different charities and asked participants to estimate whether they thought they would be more acceptable for liberals or conservatives, scored on a 7-point-scale (−3 = liberal, 3 = conservative). The final goal of the pre-study was to test the main dependent variables of donating time and money. Specifically, we wanted to estimate the proportion of participants that were willing to donate any portion of money and time. Participants were asked to imagine that they had won a £/€50 Amazon gift card. Consecutively, they were asked whether they were willing to donate money out of this £/€50 to one of the charities that had previously been described and if they were interested in volunteering for one of these charities.

Generally, results of this pre-study indicated that the estimations of our calls-to-action were in the intended directions. Specifically, the individualizing call-to-action was estimated to be substantially more in line with the Care/Harm (*M* = 5.58; *SD* = 1.50)and Fairness/Cheating (*M* = 4.88; *SD* = 1.52) foundations than was the binding call-to-action (Care/Harm: *M* = 3.92; *SD* = 2.11; Fairness/Cheating; *M* = 3.82; *SD* = 1.60). Conversely, the binding call-to-action was estimated to be substantially more in line with the Loyalty/Betrayal (*M* = 4.50; *SD* = 1.64), Authority/Subversion (*M* = 4.90; *SD* = 1.82), and Sanctity/Degradation (*M* = 3.38; *SD* = 2.02) foundations than the individualizing one (Loyalty/Betrayal: *M* = 3.32; *SD* = 1.71; Authority/Subversion: *M* = 2.72; *SD* = 1.75; Sanctity/Degradation: *M* = 2.74; *SD* = 1.70). Estimations for the neutral call-to-action were between these two estimations for each foundation (Figure C1 in Appendix C).

Furthermore, results indicated that participants mostly perceived the charities as being more appropriate for liberals than conservatives, with several charities perceived as being more or less ideologically neutral (see Figure C2 in the Appendix C). For example, *European association for cancer research, Eurochild* and *MAGmine* were perceived to be relatively neutral, while the *Group for transcultural relation* was perceived as the most liberal one. These results can be used by any researcher that decides to draw on this protocol for studying CB. Finally, 86% of the participants in the pre-study were willing to donate at least some money out of this £/€50 (*M* = 19.84; *SD* = 15.67), and 78% were interested in volunteering for one of the charities (*M* = 4.00; *SD* = 2.21).

### The Independent Variables

#### Moral Foundations

Participants’ moral foundations will be assessed using the latest version of the MFQ (Graham et al., 2008; see http://www.moralfoundations.org for more information and to access the questionnaire). The questionnaire comprises 30 items, asking participants about the extent to which different factors are relevant in their moral decision-making. Each of the five foundations is assessed with six items, and the participants’ task is to evaluate the relevance of an item or agreement with the item on a six-point scale (0 = not at all relevant/strongly disagree; to 5 = extremely relevant/strongly agree). To obtain the final score on each of the subscales, the evaluations are summed up and consecutively divided by the number of items related to the particular subscale. A score between 0 and 5 will be calculated for each of the moral foundations subscales. The internal consistencies of the subscales were reported to be α = 0.69 (Harm), α = 0.65 (Fairness), α = 0.71 (Ingroup), α = 0.74 (Authority) and α = 0.84 (Purity) (Graham et al., 2011). The MFQ is open-access and freely accessible for research purposes.

#### Moral Identity Internalization

Moral identity internalization will be measured using a five-item subscale (“Internalization” subscale) of Aquino and Reed (2002) “Moral Identity Scale”. This scale is also open access for researchers. The Internalization scale measures the degree to which moral traits are central to an individual’s self-concept. The participants’ task is to evaluate every item using a 5-point Likert scale (1 = strongly disagree; to 5 = strongly agree). The internalization scale has been shown to have good internal consistency with α = 0.73.

#### (In)congruent Calls-to-Action

We designed three types of calls-to-action with the goal of having one that is neutral, one that is congruent with liberals’ moral foundations and one that is congruent with conservatives’ moral foundations. The assumption is that most participants will be grouped into two categories: those who mostly rely on individualizing foundations, and those who mostly rely on binding foundations. Thus, two types of morally relevant calls-to-action were used. The first emphasized individualizing foundations of Care/Harm and Fairness/Cheating, whereas the second one emphasized binding foundations of Loyalty/Betrayal, Authority/Subversion and Sanctity/Degradation. The final, neutral, call-to-action was constructed without involving any moral foundations. The proposed morally (in)congruent and neutral calls-to-action are presented in Table 1. We are presenting hypothetical calls to donate to two of the charities we used in the pre-study, *Eurochild* and *City of Sanctuary*. Incidentally, one is also estimated to be more liberal (i.e., *City of Sanctuary*), while the other appears to be relatively ideologically neutral (i.e., *Eurochild;* Figure C2 in Appendix C).

**Table 1:** Calls-to-action that are going to be used in the study.

Individualizing call-to-action	**Show us your compassion** by supporting *City of Sanctuary/Eurochild* in achieving their goal *to care for refugees/protect children’s well-being*. Helping **vulnerable** *refugees/children* and **preventing suffering** is the **right thing to do**. By supporting *City of Sanctuary/Eurochild* you will get the chance to **increase fairness** and **justice and reduce harm** all over the world. Show us that you **care for others** and **make a difference** now! ^∗^In similar studies, the majority of people decided to donate some of the money should they win it. (included only in the social-norm condition).
Binding call-to-action	**Show us your integrity** by supporting *City of Sanctuary/Eurochild* in achieving their goal to *support refugees/promote children’s well-being.* **Protecting** innocent refugees/children and their families is your **civil duty**. By supporting *City of Sanctuary/Eurochild* you will get the chance to **follow proud religious and national leaders doing their obligation.** Show us that you are a **responsible community member** and **join the fight** now! ^∗^ In similar studies, the majority of people decided to donate some of the money should they win it. (included only in the social-norm condition).
Neutral call-to-action	Support *City of Sanctuary/Eurochild* in achieving their goal to *support refugees/promote children’s wellbeing*. You can help make a real difference to this very important issue. By supporting *City of Sanctuary/Eurochild* you will play an active role in finding a peaceful outcome. Show us that you are willing to support the cause now! ^∗^ In similar studies, the majority of people decided to donate some of the money should they win it. (included only in the social-norm condition).

*Bolded words represent the individualizing and binding moral frames. Italicized words pertain to the charity dealing with refugees and children, respectively.*

#### Normative Information

Normative information will either be included or omitted in addition to the calls-to-action. This normative message will read *“In similar studies, the majority of people decided to donate some of the money should they win it,”* followed by a question about the participants’ willingness to donate (see Table 1). Although the normative imessage could be stronger than the one suggested (i.e., “*In* ***this*** *study, the majority of participants decided to donate”*), it would risk being deceitful if it turned out to be inaccurate. The one we are proposing is subtler but non-deceitful, as it is based both on our pre-study results and various previous studies that showed that a substantial number of participants actually decides to donate. For example, in Nilsson et al. (2016) study, 60 and 62% of the participants donated some of the money they earned in the study, while in Reed et al. (2007) study, 90% of the participants decided to donate either time or money.

Apart from these measurements, participants will also be asked to provide some basic demographic information such as age, gender, education, nationality, ideology, and previous donations.

### The Dependent Variables

#### Attitudes Toward CB

In line with proposals for constructing TPB questionnaires (Ajzen, 2006), attitudes will be assessed with several semantic differential scales. Participants will respond to the following question: “I believe that making a donation to a described charity in terms of money or time would be:” (1) unpleasant – (7) pleasant, useful – useless, satisfying – unsatisfying, favorable – unfavorable, positive – negative, considerate – inconsiderate, pointless – worthwhile, and bad – good. Items will be scored such that higher scores indicate a more positive attitude toward CB. The internal consistency of a similar scale was shown to be α = 0.93 (Smith and McSweeney, 2007).

#### Descriptive Norms

Similarly to several previous studies (e.g., Smith and McSweeney, 2007; Croson et al., 2009; van der Linden, 2011), descriptive norms will be assessed with the following items: “How likely do you think is that people you know would donate to this charity?” (1 – very unlikely; 7 – very likely); “How much do you think an average person doing this survey would donate if they won the gift card?” (1 – nothing; 7 – all of it); “How many of the people you know would donate to this charity?” (1 – none of them; 7 – all of them); “How many of the people doing this survey would donate to this charity?” (1 – none of them; 7 – all of them). In previous studies, the reliability of similar four-item measurements of descriptive norms was found to be *p* = 0.76 (Smith and McSweeney, 2007).

#### Charitable Behavior

Since CB is not a unidimensional construct but can be classified into three main categories - helping a stranger, giving time and giving money (Charities Aid Foundation [CAF], 2017) – in this study we decided to measure CB in two different ways:

##### Time donated

The time participants are willing to donate to our charity will be assessed through participants’ interest to volunteer for the charity. Upon finishing with the questionnaires, participants will be asked the following question: “How interested are you in donating your time to volunteer for the charity?”. The question will be scored on a 7-point Likert scale (1 = not interested, 7 = very interested).

##### Money donated

As a part of the study, we will be giving three €/£50 Amazon gift cards to three random participants. Thus, each participant has an equal chance to win a €/£50 Amazon gift card. The amount of money participants are willing to donate to our partner charity will be operationalised as the amount they are willing to donate if they were to win the Amazon gift card. Therefore, participants will answer the following question: “If you were to win the €/£50 Amazon gift card, how much money out of the 50€/£would you be willing to donate to this charity?” If a participant decides to donate e.g., 20€/£, should he/she actually win the gift card, he/she would receive a 30€/£Amazon gift card, while 20€/£would actually be donated to the charity. All the measurements that will be used in this study are presented in the Appendix B.

### Design and Procedure

The procedures described in this protocol are partly based on several other studies. Specifically, when discussing the (in)congruent calls-to–action, we are following the procedures developed in the Winterich et al. (2012) and Kidwell et al. (2013) studies. Winterich et al. (2012) manipulated the description of the charities to be in line either with individualizing or binding foundations, and measured the intentions to donate a part of an Amazon $50 gift card, should the participant win it. However, unlike Winterich et al. (2012) who manipulated the charity description, we decided to manipulate the calls-to-action which is more in line with Kidwell et al. (2013) study in which they constructed individualizing and binding appeals for recycling.

Although neither of these two studies referred to attitudes and social norms, we nevertheless decided to address them in the protocol, as they seem to be important determinants of CB. Here, we draw on the methodology used in the Smith and McSweeney (2007) and van der Linden (2011) studies, but especially on Croson et al. (2009) study, who presented participants with descriptive information and measured its influence on the perception of social norms. Similarly, we are measuring both social norms and attitudes in order to gain insights not only into whether (in)congruent appeals and normative information influence donations, but also into the mechanisms of that relationship.

In sum, although the current protocol is based on several well-established procedures effectively used in previous studies, by combining them and adding several new features, we believe we managed to create a study protocol capable of yielding rich and comprehensive insights into the relationship between morality, attitudes, norms and CB.

## Stepwise Procedure

**FIGURE 2:**
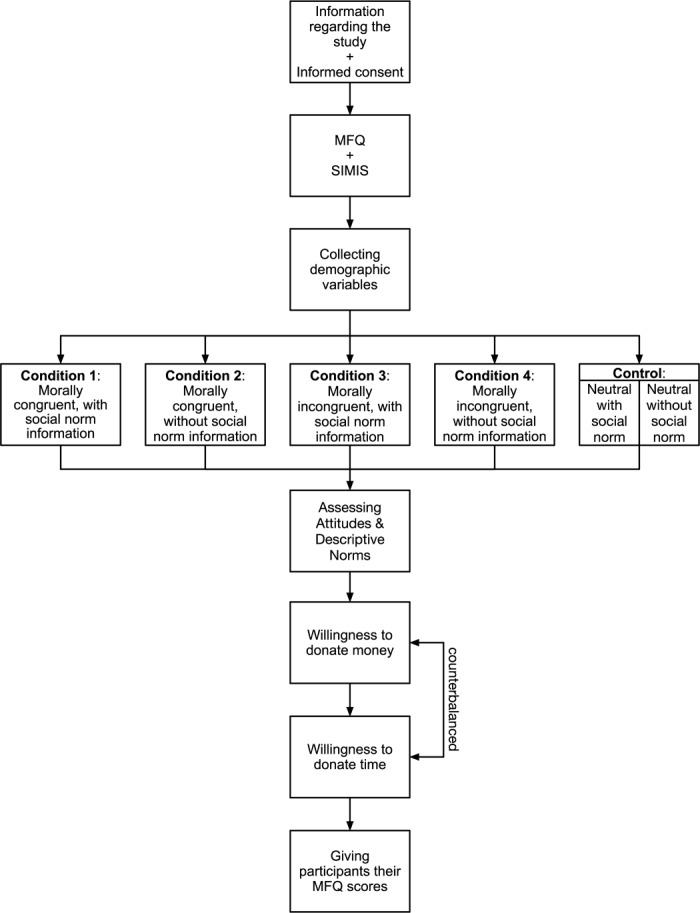
Schematic representation of the stepwise procedure.

This experimental study uses a 3 (morally (in)congruent/neutral calls-to-action) × 2 (presence/absence of social norm) between-subjects design and can be conducted online through a custom developed app or existing survey platform. The study procedure can be split into eight steps. (1) Firstly, participants receive a brief description of the study and its goals (e.g., “we want to explore the relationship between morality and CB and test the effectiveness of appeals to donate”) and are asked to provide informed consent for participation. (2) Secondly, participants complete the MFQ and the Moral Internalization subscale. (3) Thirdly, participants are asked to complete a basic demographic questionnaire. (4) Thereafter, the participants are randomly assigned to one of the two different charity causes and to one of six different experimental conditions. We use random assignment to ensure that samples are similar across conditions in terms of observed and unobserved characteristics. After the experimental exposure, participants are first asked about their attitudes toward charitable behavior and perception of social norms regarding the CB (5) and are then asked to indicate their willingness to donate (a) time and (b) money to the charity they were presented (6). The sequence of presenting the questions to donate time versus money will vary randomly in order to minimize the potential influence of sequence on both donation measures. Next, the participants are provided with a feedback regarding their scores on the MFQ and moral identity internalization questionnaire (7). Finally, an optional step is asking permission to collect participants’ Facebook data. Participants can either accept or deny this request. Regardless of their choice, in the last step, debriefing about the study and contact for further inquiries are provided (8). See Figure 2 for a visualization of this procedure.

## Statistical Analysis

To test for the effects of moral congruence of the calls-to-action, descriptive social norms, and their interaction on donations of time and money, a multivariate analysis of variance (MANOVA) will be conducted. In the same analysis, a test for the moderating effect of moral internalization on the congruence of the calls-to-action will also be conducted. This approach allows researchers to account for potential relationships between our dependent variables, and to test whether the experimental manipulations influence participants on a combination of different types of donations (Field, 2009). Furthermore, in comparison with univariate ANOVA, that requires several individual tests, using MANOVA reduces the probability of making a *Type I* error.

To test whether attitudes toward CB mediate the effects of (in)congruence of the calls-to-action on CB, and whether descriptive social norms mediate the effects of normative information on CB, a mediation analysis will be conducted. This can be done by using the bootstrapping method developed by Preacher and Hayes (Hayes, 2013). This is because previous approaches related to this analysis, such as the causal step approach (Baron and Kenny, 1986), have been criticized for lack of power, the underlying assumptions and inability to directly evaluate potential mediation (MacKinnon et al., 2002).

To determine if people’s moral foundations can be predicted from their Facebook data, various machine learning algorithms can be employed^5^. However, as this part of the study is exploratory and primarily concerned with maximizing prediction accuracy, we cannot specify the exact algorithm which will be reported. Various models and approaches can be tried out, choosing the one that exhibits the least amount of generalization error, i.e., the one that performs best on unseen data, which will be determined through cross-validation. Some possible approaches are LASSO (*Least Absolute Shrinkage and Selection Operator*) regression, multiple linear regression on clustered data and principal component regression^6^ (James et al., 2013; Kosinski et al., 2016).

The analyses can be conducted using the R language (R Core Team, 2017). For implementing the various machine learning algorithms, we will use existing packages, such as glmnet (Friedman et al., 2010), irlba (Baglama et al., 2017) and topicmodels (Grün and Hornik, 2011).

## Impact and Limitations

### Anticipated Results

Based on the theory and the literature reviewed in the introductory part, we can make some educated expectations regarding our results. First, in line with previous research (e.g., Winterich et al., 2012; Kidwell et al., 2013; Nilsson et al., 2016), we expect that our intervention in terms of morally (in)congruent calls-to-action will have a significant impact on CB. Specifically, we expect that morally congruent appeals, regardless of whether they are individualizing or binding, will foster significantly more CB compared to incongruent or neutral appeals.

Regarding the social norms, we expect that making the descriptive norms salient (“*most of the participants donate*”) will influence one’s subsequent CB. Although some of the previous studies did not find an effect of descriptive norms on CB (e.g., Smith and McSweeney, 2007; van der Linden, 2011), it seems that these studies failed to make the descriptive norms salient enough, which seems to be a prerequisite for them to be effective in influencing behavior (Cialdini et al., 1990). Thus, we hypothesize that participants who receive descriptive norms in their appeal will donate more of their money and time compared to those who do not receive a descriptive norm.

**FIGURE 3:**
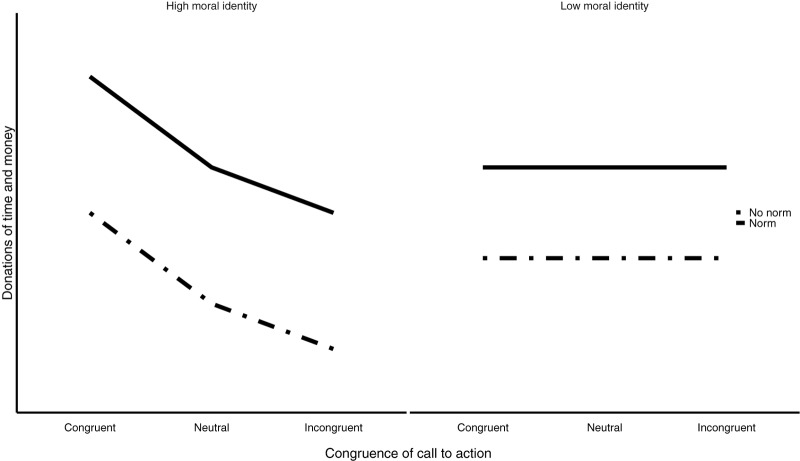
The expected effects of morally (in)congruent calls-to-action and descriptive norms on different levels of moral identity internalization.

Since we expect both morally (in)congruent calls-to-action and descriptive social norms to have an effect on CB, we hypothesize that participants receiving a morally congruent call-to-action with a descriptive norm will donate the most. Furthermore, based on previous findings regarding the influence of moral identity internalization on CB (e.g., Winterich et al., 2012), we expect to find a significant two-way interaction effect of morally (in)congruent appeals and moral identity internalization on CB. A larger effect of morally (in)congruent appeals on CB is expected for those who are high on moral identity internalization as compared to those who are low on moral identity internalization (see Figure 3).

**FIGURE 4:**
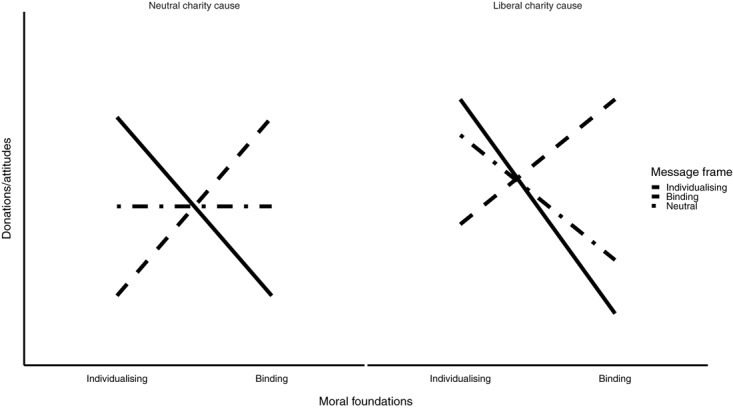
The expected effects of individualizing, neutral and binding calls-to-action on donating attitudes and CB, with respect to participants’ scores on MFQ.

We also expect to find mediating effects of attitudes and descriptive norms on the relationship between (in)congruent calls-to-action and normative messages on the one side and CB on the other. More precisely, we expect that the individualizing/binding calls-to-action will have a positive impact on donation attitudes of those participants who are high on individualizing/binding moral foundations, resulting in their willingness to donate more of their time and money. Since our second charity cause (refugee support) is already supposed to be more appealing for those high on individualizing foundations, we do not expect the individualizing calls-to-action to have a major additional impact on participants’ donating attitudes or behavior. On the other hand, we do expect that the binding calls-to-action will have a bigger impact, shifting the attitudes and increasing CB of those higher on binding moral foundations, since they are expected to have relatively negative attitudes toward such charity. See Figure 4 for a schematic representation of these effects.

### Significance of Research

We believe that the current project makes an important contribution both in theoretical and in practical terms. In theoretical terms, we hope to expand the knowledge about the scope of morally (in)congruent appeals. Rare previous research did examine the effects of the congruence between both the appeals and individual’s morality, as well as charity causes and individual’s morality. However, within the current project, we plan to explicitly contrast these two. We aim to explore whether the appeals tailored in line with one’s morality can enhance one’s donations to charitable causes, even in the case of charities for which there is no original affinity. This way, we contribute to the theoretical considerations of the role of morality in CB, but also sketch potential fundraising strategies for charities with different causes. Furthermore, another significant theoretical contribution of this project is the investigation of the role of attitudes in CB change. Specifically, within this project we not only want to show that congruent calls-to-action can have a positive impact on CB, but also to elucidate the mechanism through which this effect operates (i.e., by affecting attitudes toward CB).

Besides the effect of congruent appeals on attitudes and CB, within this project we will investigate the role of descriptive social norms in CB. Specifically, we want to test potential boundary conditions for the effectiveness of descriptive norms, especially related to their saliency. However, here we also want to go a step further and show that, if salient normative information indeed can affect CB, it probably does so through changing one’s perceptions about descriptive norms related to CB. Therefore, we believe that the current project contributes to the theoretical knowledge in several important ways.

More importantly, however, we hope that the project will have useful practical implications. Specifically, we hope to be able to provide charities with some simple yet effective tools that they could utilize in subsequent fundraising campaigns. For example, charities will be able to benefit from the often easily attainable knowledge about the approximate ideological positions of their target group by changing the way in which they deliver information about their causes and frame donation pitches. In this way, they could decrease the risk of turning away people who would potentially become their donors, by addressing them the right way. Given the low implementation costs of these interventions, we believe that charities can significantly benefit from them even if the effect sizes are modest.

Looking at the previous literature, we can expect to find small to medium effect sizes of our interventions on donating behavior. For example, Winterich et al. (2012) found small to medium effect sizes for both charitable intentions and donations (Cohen’s *d* between 0.28 and 0.46). Croson et al. (2009) found a medium impact of descriptive social norms on donations to public radio’s (Cohen’s *d* = 0.58). However, we believe that, from the perspective of charity donations, even modest effects can have big practical importance. Statistically speaking, even if our interventions exhibit only small effects on CB (Cohen’s *d* = 0.2), this still means that 58% of donations from people from the *congruent appeal + norm* intervention will be higher than the mean donation from people from the *no intervention* condition. In case we find medium effects (*d* = 0.5), as much as 69% of the *intervention* donations will be higher than the mean of the *no intervention* donations (see http://rpsychologist.com/d3/cohend/ for the visualization and interpretation of Cohen’s *d* effect sizes). Thus, taking into account the relative ease and low cost of the implementation of these interventions, we believe that any kind of improvement in terms of donations can be considered as practically significant for charity organizations.

### Limitations

Despite the potential benefits, the present project is not without limitations. Specifically, the hypothetical bias is one potential problem that is well known in the literature. Hypothetical bias is the difference between the stated and revealed value of a certain good (Murphy et al., 2005). In terms of charitable giving, people often state they are willing to donate significantly more money than they would really donate. One reason for this could be related to loss aversion which is more pronounced in case of giving real money than hypothetical money. Although we acknowledge that this is a problem, we believe that our approach at least attenuates it. Specifically, in line with the findings of Murphy et al. (2005) meta-analysis, stating that choice-based elicitation could be important in reducing the bias, we are giving our participants the choice between several different ways of allocating the 50$ Amazon card. Furthermore, we are providing real, valid gift cards from which at least some of the participants will actually donate money. Although this is still far from representing a real situation in which people are giving away their own money, we believe that it is a step away from a purely hypothetical situation in which no money would be donated. In their meta-analysis, Murphy et al. (2005) found that the median hypothetical bias fell between 1 and 1.5 (i.e., in hypothetical situations people donated between 1 and 1.5 times more than in real situations). Although impossible to know, we hope that with this approach the hypothetical bias in studies based on this protocol would not be much higher than this estimation.

However, it is important to note that, although loss aversion and hypothetical bias should affect the amount people would be willing to donate in absolute terms, it should not affect the relative differences among groups within the study. Specifically, as the participants will be randomly selected into different groups, we have no basis to believe that some groups will have significantly different hypothetical bias from others. Therefore, although absolute amounts would certainly be inflated, we believe that loss aversion and hypothetical bias should not play a significant role in our study, as the relative differences between the groups will still be observable.

A second limitation of the current study is grouping. Although we can expect the majority of our participants to fit into one of the two categories – those who rely more and those who rely less on binding foundations – some participants will not fit into this binary classification. For example, Iyer et al. (2012) showed that libertarians’ pattern of moral foundations significantly differs from those of liberals and conservatives. This means that our (in)congruent calls-to-action will not be adequately tailored toward these participants, and this may impact the overall effectiveness of our manipulations. However, most participants are expected to fit into our two categories, and those who do not will be randomly distributed between conditions. Future studies could benefit from constructing a wider range of calls-to-action to cover different types of moral foundation profiles.

Finally, we would like to address what we see as probably the most significant problem with following this protocol, and that is the Facebook app. Namely, not all researchers will be in a position to collect the data with a custom made app in order to gain access to participants’ Facebook data. However, we already noted that the part of the protocol regarding accessing Facebook data and predicting moral foundations from it is purely exploratory, and independent of the former, confirmatory part. In other words, one can reconstruct all the steps included in the confirmatory part of the protocol (i.e., presentations of the questionnaires, calls-to-action, attitudes and norms measures and dependent variables of donating time and money) using only readily available online survey tools. We believe that such a study would be easily implemented, interesting and beneficial on its own.

### Ethics

There are several important ethical concerns regarding some aspects of this protocol. First, using tailored persuasive messages in a marketing setting can yield both positive and negative outcomes. For example, it seems that people are much happier and more satisfied when spending money on things that are more congruent with their personality or needs (Matz et al., 2016). Thus, it might be expected that people will feel much better about themselves when making donations to charities whose causes and appearance are in line with their moral worldviews. However, some grimmer scenarios readily come to mind, too. Namely, if it is possible to manipulate people’s decisions and behaviors just by changing few words, this knowledge could be used for less noble purposes. For example, there are suggestions that the 2016 US presidential campaign used profiles of millions of US citizens and approached them with specifically tailored messages in order to make them stay home on the election day, possibly even swinging the election outcome in this way^7^. We fully agree that things like this one happen and will probably continue to happen. Even more, we can be sure that many companies that are not constrained by strict ethical guidelines put large amounts of money into testing different kinds of persuasive messages on a daily basis. The problem is that this mainly happens outside of user’s awareness and the knowledge gained remains private. Therefore, in our view, much more serious, ethically minded scientific research is needed in the area, because only in this way will knowledge, along with all the benefits, drawbacks and ethical concerns, enter into the public sphere and be useful for both the scientific community and the general public.

There are also certain concerns related to using data from social media to predict individual characteristics. For example, Lambiotte and Kosinski (2014) warn that it is possible for companies or individuals to use others’ social media data to infer their personal characteristics (e.g., intelligence or sexual orientation), without them noticing or without their consent. It is especially troublesome that many users may be unaware of how revealing the information available on social media can be (Kosinski et al., 2014). Youyou et al. (2015) point out that personal information can also be used to manipulate or influence people for illicit purposes. Similarly, Matz and Netzer (2017) state that social media data could be used to target individuals who are prone to impulsive or addictive behavior with ads for online casinos. These concerns are real and relevant in today’s digital societies.

However, the outlook does not have to be so bleak. Kosinski et al. (2015) argue that, for instance, Facebook users have far more control over their data than is usually assumed. According to the authors, Facebook requires its users to give consent to applications that want to use their data, and allows them to limit or revoke access to their data after it has been granted. Furthermore, users can be informed of the specific data that is being collected and the way it is being used, allowing them to make an informed decision (Kosinski et al., 2015). Also, once collected, the data can be anonymised and made unrelatable to the specific person prior to conducting analyses. We believe that these, and other steps, can be taken to make the data collection and analysis processes transparent and ethical, safeguarding the participants’ privacy.

Finally, this protocol obtained an official approval from the ethical board of the Department of Psychology, University of Zagreb. Each participant will provide consent for participation and data sharing (just the Facebook likes and no other personal information such as name, residence, friends etc.), will be familiarized with the overall goals of the study and will receive feedback detailing the exact goals of the study as well as the description and explanation of their own scores on the measures.

## Author Contributions

All authors listed have made substantial contributions to this project, and have approved the manuscript for publication. NE provided the initial idea and design for the study. All other authors contributed equally to the research design and to the preparation of this protocol.

## Conflict of Interest Statement

The authors declare that the research was conducted in the absence of any commercial or financial relationships that could be construed as a potential conflict of interest.

## Footnotes

1 Data were obtained via a Facebook app developed under the *myPersonality* project. Users had the chance to complete a personality questionnaire, an intelligence test and a satisfaction with life questionnaire. Various demographic attributes were collected from users’ Facebook profiles and some characteristics were obtained through a survey (Kosinski et al., 2013). Users could also volunteer their social media data - specifically, Facebook *likes* – for research purposes.

2 http://www.pewinternet.org/2018/05/31/teens-social-media-technology-2018/

3 https://en.wikipedia.org/wiki/Facebook/%E2/%80/%93Cambridge_Analytica_data_scandal

4 http://moralfoundations.org/questionnaires

5 The first step is usually data preprocessing, which also includes trimming down the data set (i.e., removal of non-informative entries; e.g., a Facebook page that is *liked* by less than 150 users; Kosinski et al., 2016). After that, a form of dimensionality reduction is usually applied to the data (Kosinski et al., 2013, 2016). This is done because analyzing high-dimensional data sets can exacerbate issues such as model overfitting or multicollinearity (James et al., 2013). In the end, machine learning algorithms are trained in order to identify or predict users’ characteristics.

6 For example, Youyou et al. (2015) used LASSO regression when judging participants’ scores on the Big Five dimensions. *LASSO* is an approach to regression problems that applies penalties for the number of variables in a model, and which can shrink certain variables’ coefficients to zero, thus serving as a variable selection technique (James et al., 2013). Kosinski et al. (2013) first conducted a singular value decomposition on their dataset in order to reduce its dimensionality, and then trained linear and logistic regression models to predict users’ characteristics.

7 https://motherboard.vice.com/en_us/article/mg9vvn/how-our-likes-helped-trump-win
