# Supplementary material for: The Effect of Moral Congruence of Calls to Action and Salient Social Norms on Online Charitable Donations: A Protocol Study
Source: Front Psychol. 2018 Oct 26;9:1913. doi: 10.3389/fpsyg.2018.01913 (PMC6212564; doi:10.3389/fpsyg.2018.01913)
Supplement: Supplementary file 1 [file Data_Sheet_1.pdf]

Appendix A

Table A.
Potential Facebook groups to target

**Country**	**Facebook pages** **Liberal**	**Conservative**
**UK**	*Young Labour*<br>*Young Liberals*	*UK Young Conservatives*<br>*Young Unionists*
**Netherlands**	*Liberale vrienden*<br>*De Haagse VVD*	*Conservatief Café*<br>*PerspectieF, ChristenUnie-jongeren*
**Germany**	*"Schwulen Gruppe" - Ausländer & Deutsche*<br>*SCHWULE FAMILIE*	*Gegenwind Deutschland*<br>*Die ÖKO Lüge*
**Italy**	*Articolo Uno- Movimento Democratico e Progressista*	*Movimento Giovani Padani*

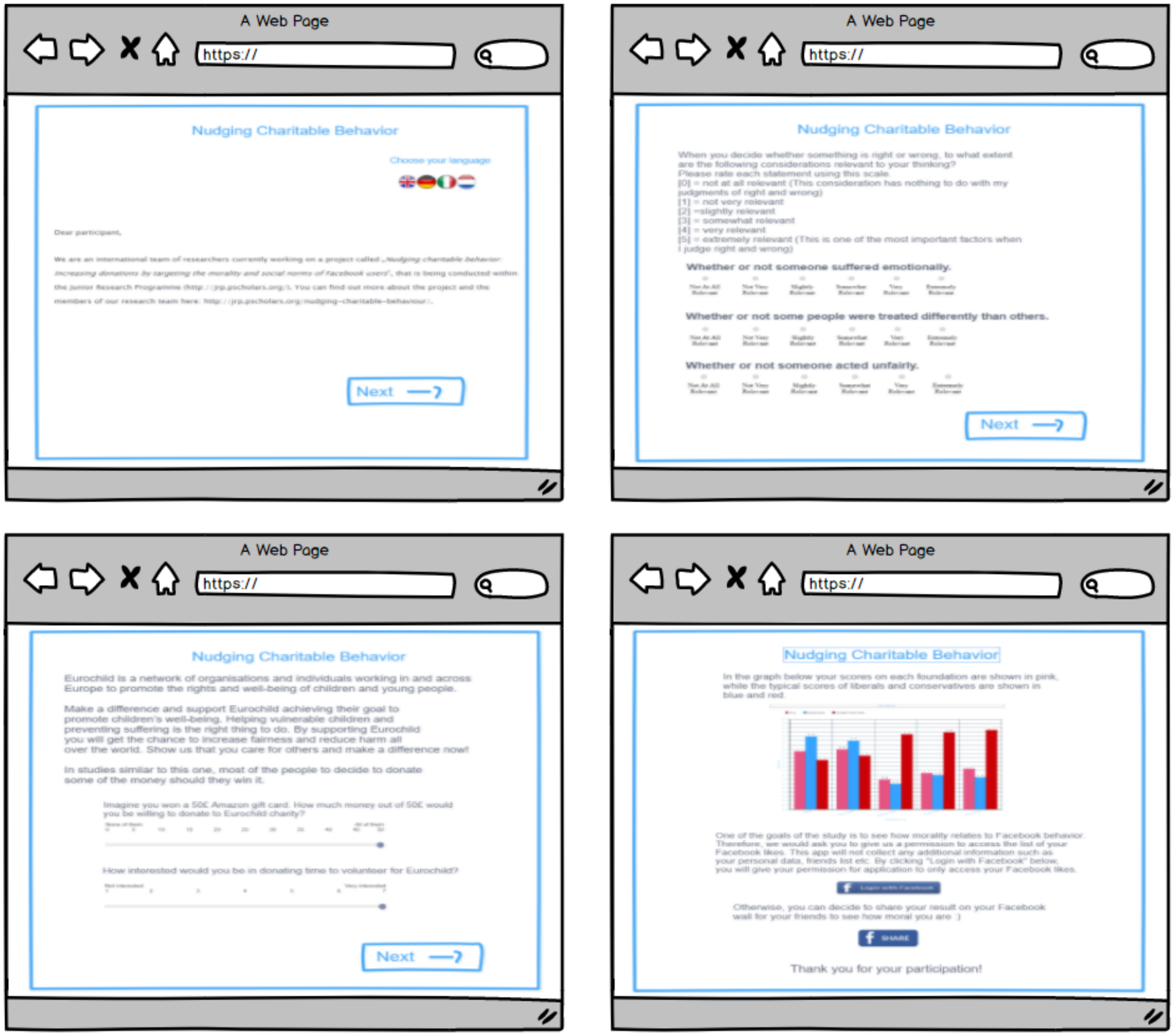

Figure A. The wireframe of a Facebook app

Appendix B

Instruments to be used in the study

1) Moral foundations questionnaire (Graham, Haidt, & Nosek, 2008)

Part 1. When you decide whether something is right or wrong, to what extent are the following considerations relevant to your thinking? Please rate each statement using this scale:

[0] = not at all relevant (This consideration has nothing to do with my judgments of right and wrong)

[1] = not very relevant

[2] = slightly relevant

[3] = somewhat relevant

[4] = very relevant

[5] = extremely relevant (This is one of the most important factors when I judge right and wrong)

1. Whether or not someone suffered emotionally
2. Whether or not some people were treated differently than others
3. Whether or not someone’s action showed love for his or her country
4. Whether or not someone showed a lack of respect for authority
5. Whether or not someone violated standards of purity and decency
6. Whether or not someone was good at math
7. Whether or not someone cared for someone weak or vulnerable
8. Whether or not someone acted unfairly
9. Whether or not someone did something to betray his or her group
10. Whether or not someone conformed to the traditions of society
11. Whether or not someone did something disgusting
12. Whether or not someone was cruel
13. Whether or not someone was denied his or her rights
14. Whether or not someone showed a lack of loyalty
15. Whether or not an action caused chaos or disorder
16. Whether or not someone acted in a way that God would approve of

Part 2. Please read the following sentences and indicate your agreement or disagreement:

[0] Strongly disagree

[1] Moderately disagree

[2] Slightly disagree

[3] Slightly agree

[4] Moderately agree

[5] Strongly agree

17. Compassion for those who are suffering is the most crucial virtue.
18. When the government makes laws, the number one principle should be ensuring that everyone is treated fairly.
19. I am proud of my country's history.
20. Respect for authority is something all children need to learn.
21. People should not do things that are disgusting, even if no one is harmed.
22. It is better to do good than to do bad.
23. One of the worst things a person could do is hurt a defenseless animal.
24. Justice is the most important requirement for a society.
25. People should be loyal to their family members, even when they have done something wrong.
26. Men and women each have different roles to play in society.
27. I would call some acts wrong on the grounds that they are unnatural.
28. It can never be right to kill a human being.
29. I think it's morally wrong that rich children inherit a lot of money while poor children inherit nothing.
30. It is more important to be a team player than to express oneself.
31. If I were a soldier and disagreed with my commanding officer's orders, I would obey anyway because that is my duty.
32. Chastity is an important and valuable virtue.

2) **Moral identity internalization** (Aquino & Reed, 2002)

Listed below are some characteristics that may describe a person:
Caring, Compassionate, Fair, Friendly, Generous, Hardworking, Helpful, Honest, Kind
The person with these characteristics could be you or it could be someone else. For a moment, visualize in your mind the kind of person who has these characteristics. Imagine how that person would think, feel, and act. When you have a clear image of what this person would be like, answer the following questions using a 5-point Likert scale (1 strongly disagree, 5 strongly agree).

1. It would make me feel good to be a person who has these characteristics.
2. Being someone who has these characteristics is an important part of who I am.
3. I would be ashamed to be a person who has these characteristics.
4. Having these characteristics is not really important to me.
5. I strongly desire to have these characteristics.

3) **Attitudes towards charitable behavior**

I believe that making a donation to a described charity in terms of money or time would be:

(1) unpleasant – (7) pleasant

(1) useful – (7) useless

(1) satisfying – (7) unsatisfying

(1) favorable – (7) unfavorable

(1) positive – (7) negative

(1) considerate – (7) inconsiderate

(1) pointless – (7) worthwhile

(1) bad – (7) good.

4) Descriptive social norms

1) How likely do you think is that people you know would donate to this charity?

(1 – very unlikely; 7 – very likely)

2) How much do you think an average person doing this survey would donate if they won the gift card?

(1 – nothing; 7 – all of it)

3) How many of the people you know would donate to this charity?

(1 – none of them; 7 – all of them)

4) How many of the people doing this survey would donate to this charity?

(1 – none of them; 7 – all of them)

5) Charitable behavior

The participants will answer the following questions:

"If you were to win the €/£50 Amazon gift card, how much money out of the 50€/£ would you be willing to donate to this charity?"

€/£0 €/£5 €/£10 €/£15 €/£20 €/£25 €/£30 €/£35 €/£40 €/£45 €/£50

"How interested are you in donating your time to volunteer for the charity?"

1	2	3	4	5	6	7
Not interested at all						Very interested

Appendix C

Pre-study results

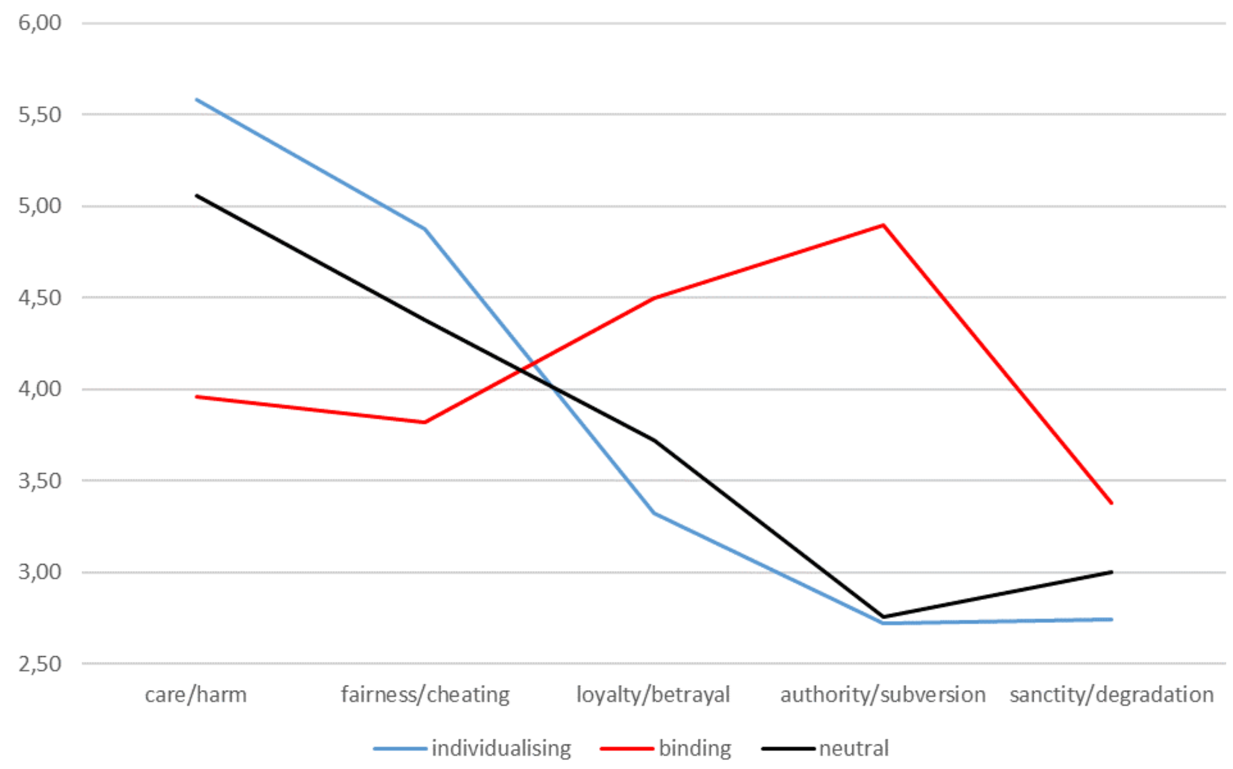

Figure C1. Estimations of the congruence of calls-to-action and moral foundations.

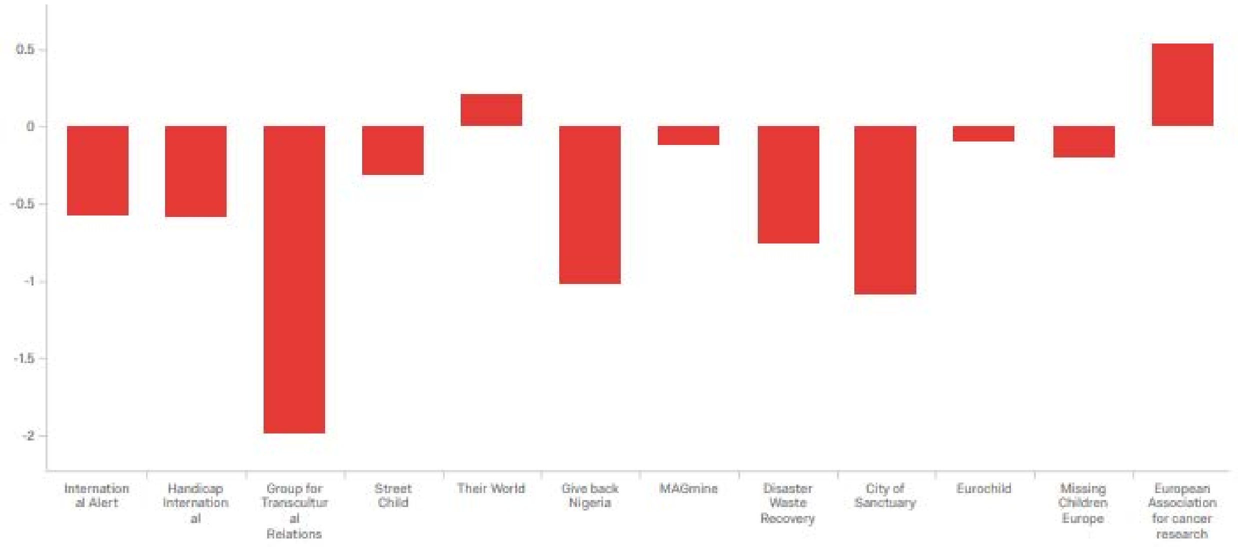

Figure C2. Mean estimations of appropriateness of different charitable organizations for liberals and conservatives (7 point scale where -3 = appropriate for liberals and +3 = appropriate for conservatives).
